# Characteristics of steroid hormones in systemic lupus erythematosus revealed by GC/MS-based metabolic profiling

**DOI:** 10.3389/fendo.2023.1164679

**Published:** 2023-07-27

**Authors:** Dehong Wu, Lingxia Ye, Xiafeng Zhang, Mengdi Yin, Yixuan Guo, Jia Zhou

**Affiliations:** ^1^ Department of Rheumatology, The Second Affiliated Hospital of Zhejiang Chinese Medical University, Hangzhou, Zhejiang, China; ^2^ Department of Endocrinology and Metabolism, The Second Affiliated Hospital, Zhejiang University School of Medicine, Hangzhou, Zhejiang, China; ^3^ Institute of Basic Research in Clinical Medicine, College of Basic Medical Sciences, Zhejiang Chinese Medical University, Hangzhou, Zhejiang, China

**Keywords:** hormone, metabolic profiling, SLE, GC/MS, steroid

## Abstract

**Background:**

Systemic lupus erythematosus (SLE) is a systemic autoimmune disease with a remarkable predominance in female, suggesting that steroid hormones may be involved in the pathogenesis. However, steroid signature of SLE patients has not been fully explored.

**Methods:**

A metabolic profiling analysis based on gas chromatography/mass spectrometry (GC/MS) with high sensitivity and reproducibility was employed to comprehensively reveal SLE-specific steroid alterations.

**Results:**

More than 70 kinds of steroids in urine were detected by gas chromatography/mass spectrometry (GC/MS) to reveal SLE-specific steroid alterations. Principle component analysis demonstrated that the steroid profile was obviously distinguished between patients with SLE and controls. A lower level of total androgens was observed in patients, and nine androgens [dehydroepiandrosterone (DHEA), testosterone, Etio, androsterone, βαβ-Diol, Epi-An, Epi-DHT, 16α-OH-DHEA, and A-Diol] underwent significant decrease. Moreover, patients with SLE exhibited a slightly higher level of total estrogens than controls, and three estrogens (17-Epi-E3, 17α-E2, and E3) were remarkably increased. Furthermore, we identified the elevation of two sterols (Lan and Chol), and the reduction of one corticoid (11-DeoxyF) and two progestins (5α-DHP and 11β-OH-Prog) in patients.

**Discussion:**

In this study, metabolic signature of urinary steroids associated with SLE was comprehensively defined by GC/MS for the first time, and steroid metabolism disorders were found in patients with SLE, especially the conversion of androgens to estrogens. Our findings will provide new insights for a deeper understanding of the mechanism of steroid hormones in the pathogenesis of SLE and will help to unravel the reason of sexual disparity in SLE.

## 1 Introduction

Systemic lupus erythematosus (SLE) is a systemic autoimmune disease, characterized by T-cell dysfunction, abnormal B-cell activation, and autoantibody production. The clinical manifestations of SLE are complex and diverse, and most patients will gradually develop multiple-organ damage, among which renal damage is more prominent. The etiologies and mechanisms of the disease have not been fully understood. The incidence rate of SLE is about 70/100,000 in China and is increasing year by year. The occurrence of SLE has a remarkable predominance in women, with a 7–9:1 women-to-men ratio and is much more common in women of childbearing age (1), suggesting that, in addition to factors such as heredity, environment, and infection, hormones may also participate in the pathogenesis of SLE (2).

Some studies based on murine models or humans have suggested an association between steroid hormones and lupus. Recent clinical trials found that oral contraceptives containing estrogen activated the disease, but the use of contraceptives containing only progesterone can reduce the risk of SLE (3). Patients with SLE exacerbated with pregnancy, especially in terms of renal lupus (4). In female lupus-prone mice, estrogen supplementation can worsen the disease, whereas gonadectomy or androgens supplementation can ameliorate the disease; in addition, the removal of the gonads in male lupus-prone mice increases disease susceptibility (5, 6).

Numerous studies verified that hormones participate in the regulation of immune system, which might be one of the mechanisms behind. Estrogen, androgen, progestin, corticoid, and sterol are the main types of steroid hormones in the body. Different types of steroid hormones play different roles in the immune system, including the development, homeostasis, activation, and differentiation of lymphocytes and the production of cytokines, therefore impacting the immune responses differentially (7). Estrogen targeted a serious of immune cells, including T lymphocytes, B lymphocytes, mononuclear macrophages, and natural killer cells, which were all proven as important roles in the pathogenesis of SLE (8–10). Similarly, progesterone can also act on the above-mentioned various immune cells to exert an immunomodulatory effect (7). Androgens can reduce the proliferation and differentiation of lymphocytes and may inhibit the production of immunoglobulins (11), which is generally regarded as beneficial for autoimmune diseases.

Endogenous steroid hormones are usually generated in the adrenal cortex, ovaries, and testes, which are mainly produced by the metabolism of cholesterol through a series of enzymes. There are mutual transformations between various steroids in the body, which are constantly produced or consumed, maintaining in a dynamic equilibrium state. Disorders in steroid metabolism may influence the occurrence and development of SLE through immune dysfunction. Hence, exploring the expression characteristics of steroids in patients with SLE is of great value for the in-depth study of the pathological mechanism, diagnosis, and treatment of SLE. Previous studies have found abnormalities in hormones in patients with SLE, although these studies are mainly aimed at several common steroid hormones, such as estradiol (E2), testosterone, and dehydroepiandrosterone (DHEA) (12–14). However, there are dozens of steroids in the body, so it is necessary to perform a global analysis of the steroid profile in patients with SLE to better understand the correlation between SLE and hormones.

Major technologies for current monitoring hormone levels include radioimmunoassay, direct immunoassay, and mass spectrometry (MS)–based assay. Among these, radioimmunoassay and direct immunoassay are most widely used. Several studies comparing different assays indicated the discrepancies that the lack of precision and accuracy at low hormone concentrations makes immunoassays unreliable for monitoring patients with hypogonadism (15–18). Thus, MS-based assays become the gold standard for steroid hormones detection (19). Beyond that, MS-based assays can detect much more kinds of steroids than immunoassays. MS-based assays refer to gas chromatography (GC)/tandem MS and liquid chromatography (LC)/tandem MS. However, until now, there is no any paper reporting the steroid profile changes in patients with SLE with the method of MS-based assays.

To comprehensively characterize the SLE-specific metabolism of steroids, this study intends to employ a highly sensitive metabolic profiling analysis based on GC/MS to detect the changes in steroid hormones in the urine of SLE female patients, which might be helpful to deepen the understanding of the role of hormones in the pathogenesis of SLE and provide new ideas for the diagnosis and treatment of SLE.

## 2 Materials and methods

### 2.1 Subjects

This cross-sectional study enrolled 15 patients with SLE and 15 age-matched healthy volunteers at The Second Affiliated Hospital of Zhejiang Chinese Medical University between 1 January 2018 and 31 December 2019. All participants were women, 26 to 55 years old, not pregnant at the time. They all completed a screening evaluation that included a detailed medical history. These patients with SLE were diagnosed according to the 1997 American College of Rheumatology (ACR) Classification criteria and are under anti-SLE treatment, whereas the healthy volunteers were absent of any clinical manifestations of SLE or any inflammatory or autoimmune diseases. This study was approved by the ethics committee of The Second Affiliated Hospital of Zhejiang Chinese Medical University (No. AF-BG-006-1.0), and every subject has given an informed consent.

### 2.2 Reagents

The internal standards (d3-testosterone, d4-cortisol, d9-progesterone, d7-cholesterol, 13c3-androstene-3,17-dione, and 13c3-estrone) were purchased from Cerilliant (Round Rock, TX, USA). L-ascorbic acid, sodium acetate, acetic acid, β-glucosaldosidase/arylsulfatase, ammonium iodide (NH4I), dithioerythritol (DTE), and N-methyl-n-(trimethylsilyl)trifluoroacetamide (MSTFA) were purchased from Sigma-Aldrich (St. Louis, MO, USA). Commercial standards of steroids (dehydroepiandrosterone, testosterone, 11-deoxycorticosterone, cortisone, cortisol, 16α-hydroxyestrone, pregnenolone, 17α-hydroxypregnenolone, epipregnanolone, dihydrotestosterone, 21-hydroxyprogesterone, androsterone, epiandrosterone, corticosterone, estrone, 17β-estradiol, estriol, 16-epiestriol, progesterone, 17α-hydroxyprogesterone, desmosterol, cholesterol, etc.) were purchased from Sigma-Aldrich, J&K Chemical Ltd. (Beijing, China), or Santa Cruz Biotechnology, Inc. (Santa Cruz, CA). Chromatographic pure hexane, methanol, and ethyl acetate were purchased from Merck (Fairfield, OH, USA). The Oasis HLB SPE cartridge was obtained from Waters (1.5 ml, 60 mg; Waters, Milford, MA, USA).

### 2.3 Sample collection and preparation for steroid profiling analysis

The analysis of urinary steroid profile was based on the protocol of Moon (20). Urine (10 ml) was collected from subjects between 6:00 and 8:00 in the morning, after fasting for 12 h. A total of 2 ml of urine was added into a microcentrifuge tube containing 20 μl of internal standard solution (d3-testosterone, 10 μg/ml; d4-cortisol, 35 μg/ml; d9-progesterone, 35 μg/ml; d7-cholesterol, 35 μg/ml; 13c3-androstene-3,17-dione, 10 μg/ml; and 13c3-estrone, 10 μg/ml). Before extraction and purification of steroids by SPE, the SPE cartridges were activated and balanced with 2 ml of methanol and 2 ml of pure water. Subsequently, the sample was loaded and washed with high pure water twice. Then, the steroids were eluted twice with 2 ml of methanol. The collected eluents were dried with a small stream of nitrogen. Acetate buffer (1 ml; 0.2 M, pH 5.2), 0.2% L-ascorbic acid (100 μl), and glucosaldehydase/arylsulfatase (50 μl) were added and then incubate at 55°C for 3 h. The solution was extracted twice with ethyl acetate/n-hexane (2:3, v/v). The upper layer were merged and concentrated to dry under nitrogen at 40°C and further dried in a vacuum dryer for 60 min. Before GC/MS analysis, 40 μl of MSTFA/NH4I/DTE mixture (500:4:2, v/w/w) was added for derivation and reacted for 20 min at 60°C in water bath. Equal amounts of urine samples were mixed as quality control (QC) samples and then subjected to the same treatment as described above and analyzed together with the actual samples.

### 2.4 Standard solution preparation

A series of reference standards were selected to generate steroid mass spectral library to explore the mass fragmentation characteristics of different kinds of steroids and support the structural identification of steroids in urine. Commercial steroid standards were prepared as stock solutions at a concentration of 100 μg/ml in methanol and stored at −20°C. Prior to analysis, the standard solutions were diluted to 0.1–10 μg/ml and lyophilized in a centrifugal concentrator. After derivatization using the method mentioned in Section 2.3, the mass spectra of each steroid standard were obtained by GC/MS.

### 2.5 GC/MS analysis

GC/MS analysis was performed on an Agilent 7890/5975C GC/MS (Agilent Technologies, Santa Clara, CA, USA). Steroids were separated using a 25 mm × 0.2 mm × 0.33 μm Ultra-1 column (J&W Scientific, Folsom, CA, USA). The sample injection volume was 2 μl with a split ratio of 5:1. Helium (99.9996%) was used as the carrier gas, and the flow rate was 1.0 ml/min. The oven temperature program was as follows: the initial temperature was 215°C, ramped to 260°C at 1°C/min and then ramped to 320°C at 15°C/min, and held for 5 min. The injector and transfer line temperatures were both 280°C. Full scan mode and selective ion scan were used for qualitative and quantitative analysis of urinary steroids, respectively. During the analysis process, different groups of samples were interspersed, and one QC sample was added to the analysis sequence for every five samples.

### 2.6 Data processing and statistical analysis

The structure of urinary steroid was identified by comparing the acquired mass spectra and retention indices with our self-constructed steroid mass spectral library, commercial National Institute of Standards and Technology (Boulder, CO, USA) library, and mass spectra published in previous references (20, 21). The peak area of each steroid was integrated by Agilent GC/MS workstation (Agilent Technologies, Santa Clara, CA, USA), and the quantitative ion was determined according to the standards or reference (21). After normalized to the internal standard of corresponding steroid species, the data were subjected to a principal component analysis (PCA) using the SIMCA-P 11.0 version (Umetrics AB, Umea, Sweden). PCA, as an unsupervised learning technique, is employed to reduce the dimensionality of complex datasets and identify the most significant variations within the data. By applying PCA, we can gain an overview of the disparities in steroid profiles between the SLE and control groups. Furthermore, orthogonal signal correction (OSC) partial least-squares discriminant analysis (PLS-DA) was performed to distinguish patients with SLE and controls and to screen the different steroids between groups. OSC PLS-DA is a supervised multivariate statistical analysis method that filters out irrelevant variations to classification and can more effectively capture the difference information between groups as compared to PCA. Analysis of variance testing of the cross-validated residuals (CV-ANOVA) was used to measure the reliability of the OSC PLS-DA model. VIP (variable importance in projection) values indicate the contribution of each variable to the distinction between groups in the OSC PLS-DA model. A high VIP value suggests that the corresponding variable is important in distinguishing between groups, whereas a low VIP value indicates a low correlation. Here, variables with VIP > 1.0 were selected as potential discriminant steroids. SPSS 18.0 (International Business Machines Corp., Armonk, USA) was employed to conduct T-test for each steroid. OSC PLS-DA (VIP > 1) and T-test (P < 0.05) were combined to find out the differential steroids between two groups. On the basis of the screened steroids, the pathway analysis was performed in the MetaboAnalyst website (http://www.metaboanalyst.ca), and the involved metabolic pathways were visualized by using Metscape 3.1.3. Furthermore, Pearson analysis was carried out to measure the correlation between the discriminant steroids and SLE disease activity index (SLEDAI).

## 3 Results

### 3.1 Clinical characteristics of participants

All subjects were women, and participants in both groups were matched in age (p > 0.05). Patients with SLE participating in the study fulfilled the ACR revised criteria for the classification of SLE and received anti-SLE treatment. The clinical characteristics of participants are listed in **Supplementary Materials** (**Table S1**). All patients with SLE showed positive antinuclear antibodies, and seven of them had kidney damage. None of the recruited subjects were taking any contraceptives, sex hormones, or related medicine.

### 3.2 Metabolic profiles of steroids in patients with SLE

**Figure 1:**
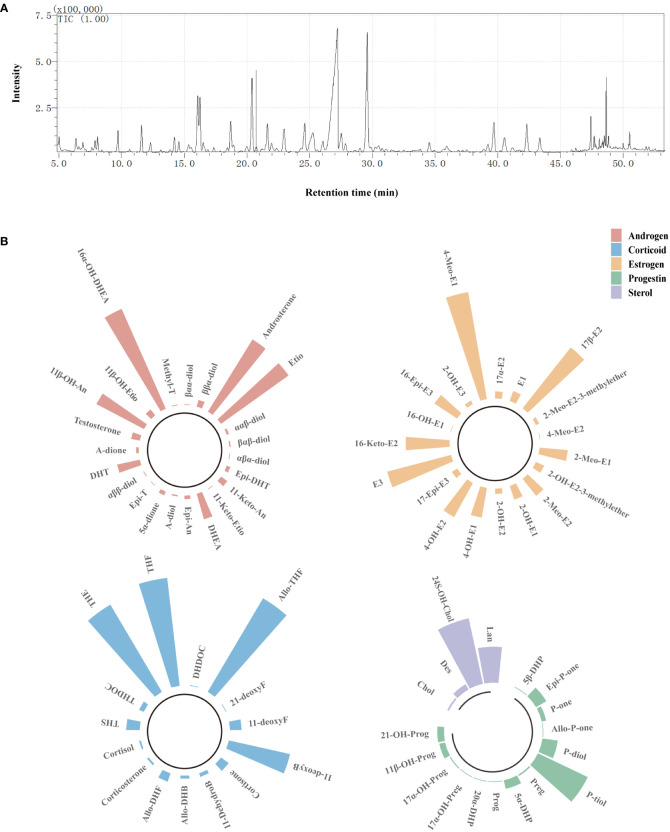
Detection of urinary steroids using GC/MS. **(A)** Steroid profile of QC sample. **(B)** Circular bar charts showing the composition of each steroid species. Bar represents the proportion of individual steroids in the corresponding steroid species, and the length of bar was determined on the basis of the average proportion of that steroid in all samples. Red bars refer to androgens, blue bars refer to corticoids, orange bars refer to estrogens, green bars refer to progestins, and purple bars refer to sterols.

Seventy-five kinds of steroids were detected in urine samples, including 23 kinds of androgens, 15 kinds of corticoids, 19 kinds of estrogens, 14 kinds of progestins, and four kinds of sterols (**Figure 1**). The full names and abbreviations of steroids are listed in **Supplementary Materials** (**Table S2**). Circular bar charts were used to display the composition of each steroid species, and the proportion of individual steroids in the corresponding steroid species was calculated and averaged over all samples (**Figure 1B**). As shown in **Figure 1**, the most abundant androgens was 16α-OH-DHEA, followed by Etio and androsterone; THE (tetrahydrocortisone), Allo THF (allotetrahydrocortisol), and THF were the three most abundant corticoids; among all estrogen, E3 and 17β-E2 had the highest abundance; in addition, P-tiol (Pregnanetriol) and 24S-OH-Chol (24S-Hydroxycholesterol) depicted a higher abundance.

### 3.3 Overall differences in steroids between patients with SLE and controls

**Figure 2:**
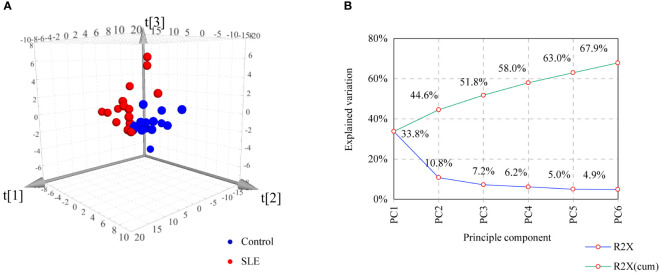
PCA of controls and patients with SLE based on urinary steroid profiles. **(A)** Score plot. **(B)** Scree plot showing the percentage of variance explained by individual principal component.

To assess the general differences of steroid profiles between patients with SLE and controls, unsupervised PCA analysis was performed on steroid data after UV scaling. As shown in **Figure 2**, PCA score plot based on the first three principle components (R2X = 51.8%) demonstrated a tendency of separation between the SLE group and the control group, indicating an alteration in steroid hormone metabolism in patients with SLE.

### 3.4 Alterations in the total amount of steroids

**Figure 3:**
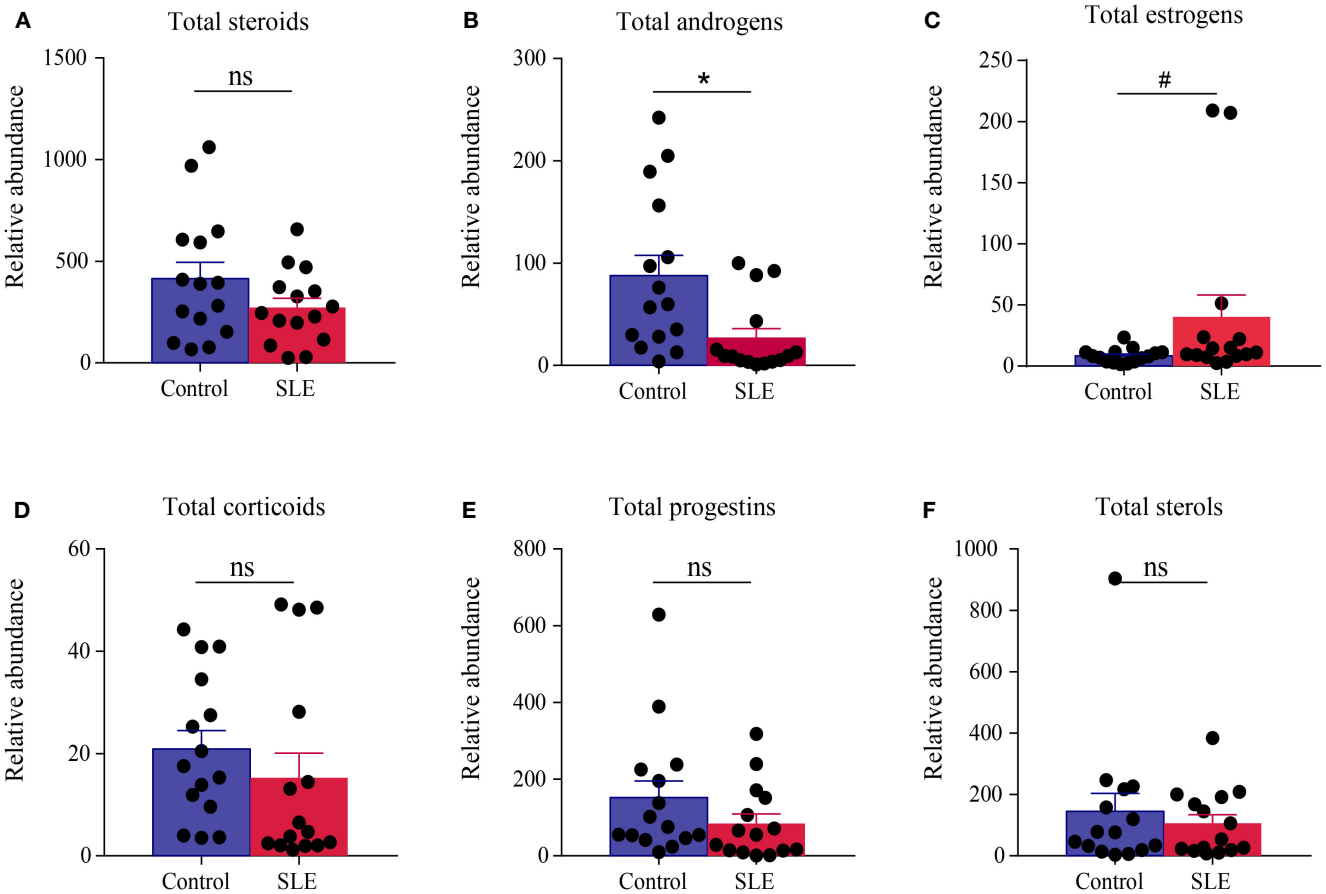
Comparison of the total amount of various steroids in the urine of the control group and patients with SLE. **(A)** Total steroids, **(B)** total androgens, **(C)** total estrogens, **(D)** total corticoids, **(E)** total progestins, and **(F)** total sterols. * indicates a significant difference (P < 0.05), # indicates a slight difference (P < 0.1), and ns indicates no statistical significance (P > 0.1).

The levels of total steroids, total androgens, total estrogens, total corticoids, total progestins, and total sterols in urea samples were measured for each participant. Compared with the control group, there were no significant changes in the levels of total steroids, total corticoids, total progestins, and total sterols in the SLE group. Significantly, there was a giant decrease in the level of total androgens in patients with SLE (P < 0.05), and there was a slight trend of elevation in total estrogens (P < 0.1, **Figure 3**).

### 3.5 Screening of altered steroid species in patients with SLE

**Figure 4:**
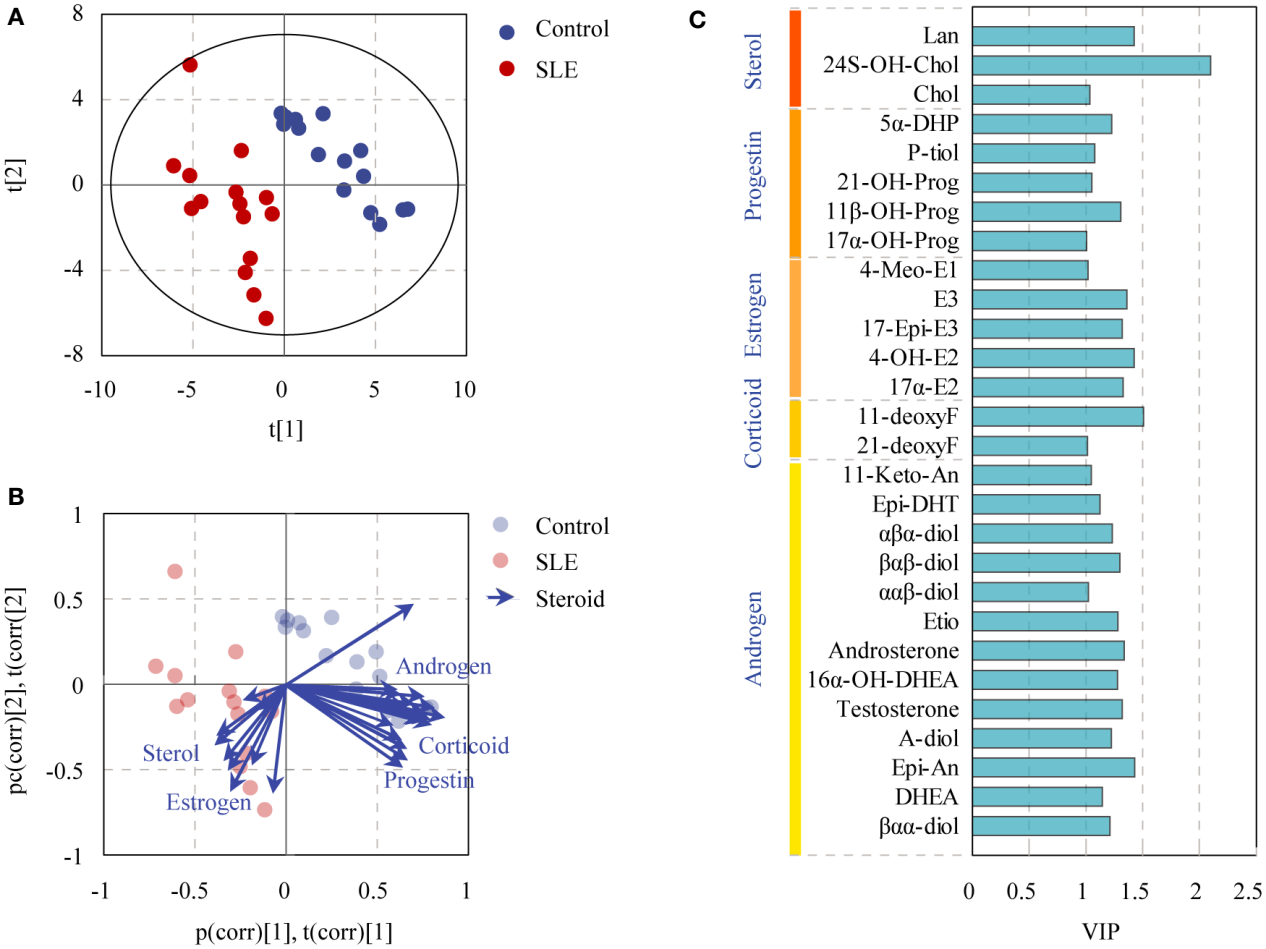
OSC PLS-DA of controls and patients with SLE based on urinary steroid profiles. **(A)** OSC PLS-DA score plot, **(B)** biplot displaying co-chart scores and loadings, and **(C)** steroids with VIP > 1.

To reveal the specific alterations of steroids in patients with SLE, PLS-DA was conducted to compare the steroid profiles between two groups after OSC filtration. The model matched well [R2Y(cum) = 0.932 and Q2Y(cum) = 0.799]. CV-ANOVA test was carried out for significance testing of OSC PLS-DA model, and the P-value of CV-ANOVA was less than 0.05, suggesting that the model was significant. The score plot showed that the steroid profiles of patients with SLE were obviously distinguished from that of the control group (**Figure 4A**). More interestingly, the biplot displaying co-chart scores and loadings showed that some kinds of androgens, corticoids, and progestins tended to be decreased; meanwhile, some kinds of sterols and estrogens seemed more enriched in patients with SLE (**Figure 4B**). The steroids with VIP>1 were listed in **Figure 4C** based on the OSC PLS-DA model.

**Figure 5:**
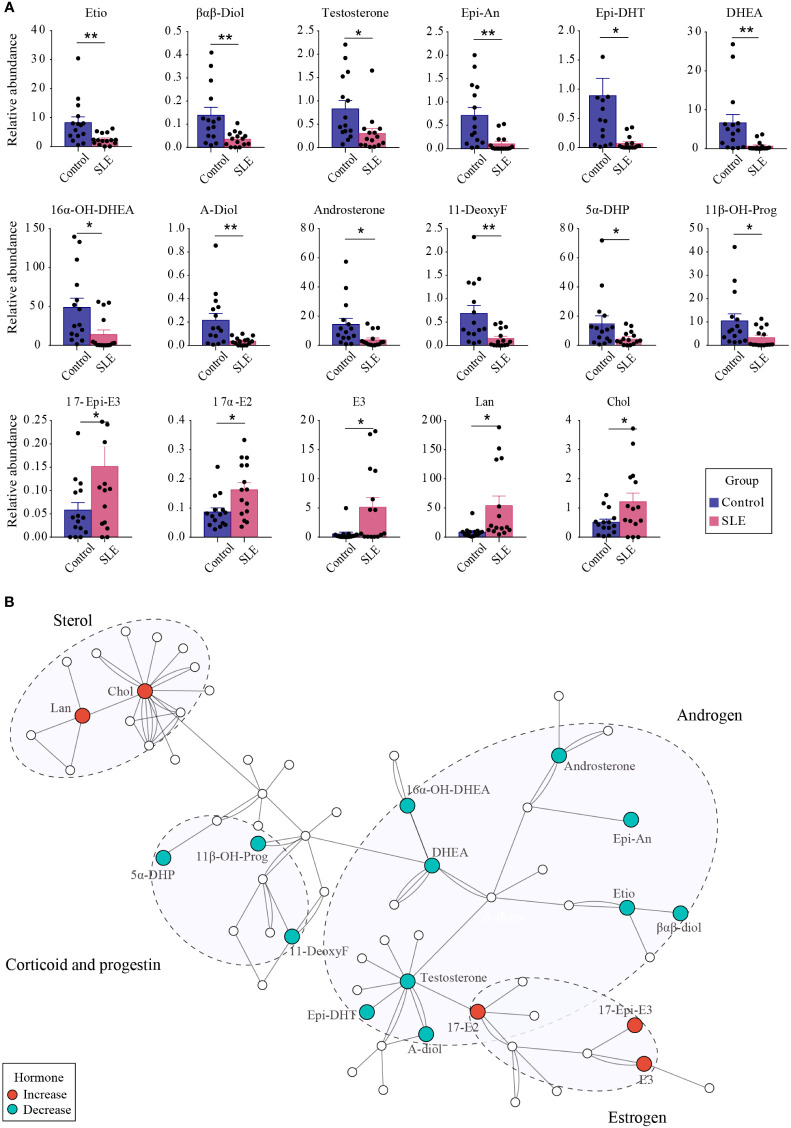
The difference of urinary steroids between the control group and patients with SLE. **(A)** Bar plot showing the changing trend of 17 different steroids between the two groups. ** indicates P < 0.01 between groups, and * indicates P < 0.05 between groups. **(B)** Pathway analysis of steroids that were altered in SLE patients. Red dots indicate significantly increased steroids in patients with SLE, whereas green dots indicate significantly reduced steroids.

Differential steroids between patients with SLE and healthy controls were screened according to the VIP values (VIP>1) of the OSC PLS-DA model and P-values of univariate analysis (P < 0.05). A total of 17 kinds of differential steroids were screened, of which none kinds of androgens (Etio, βαβ-Diol, testosterone, Epi-An, Epi-DHT, DHEA, 16α-OH-DHEA, A-Diol, and androsterone), one kind of corticoid (11-DeoxyF), and two kinds of progestins (5α-DHP and 11β-OH-Prog) were significantly decreased in patients with SLE, whereas three kinds of estrogens (17-Epi-E3, 17α-E2, and E3) and two kinds of sterols (Lan and Chol) showed significant increases in patients with SLE (**Figure 5A**).

Through metabolic pathway analysis, we found that the different steroids found in urine samples from patients with SLE and healthy controls were mainly enriched in biological processes such as steroidogenesis, androstenedione metabolism, and steroid biosynthesis (**Figure 5B**), suggesting the presence of steroid metabolism disorders in patients with SLE. In particular, the conversion of androgens to estrogens was promoted, which led to abnormal levels of androgens and estrogens.

### 3.6 Correlation between the differential steroids and SLEDAI

**Figure 6:**
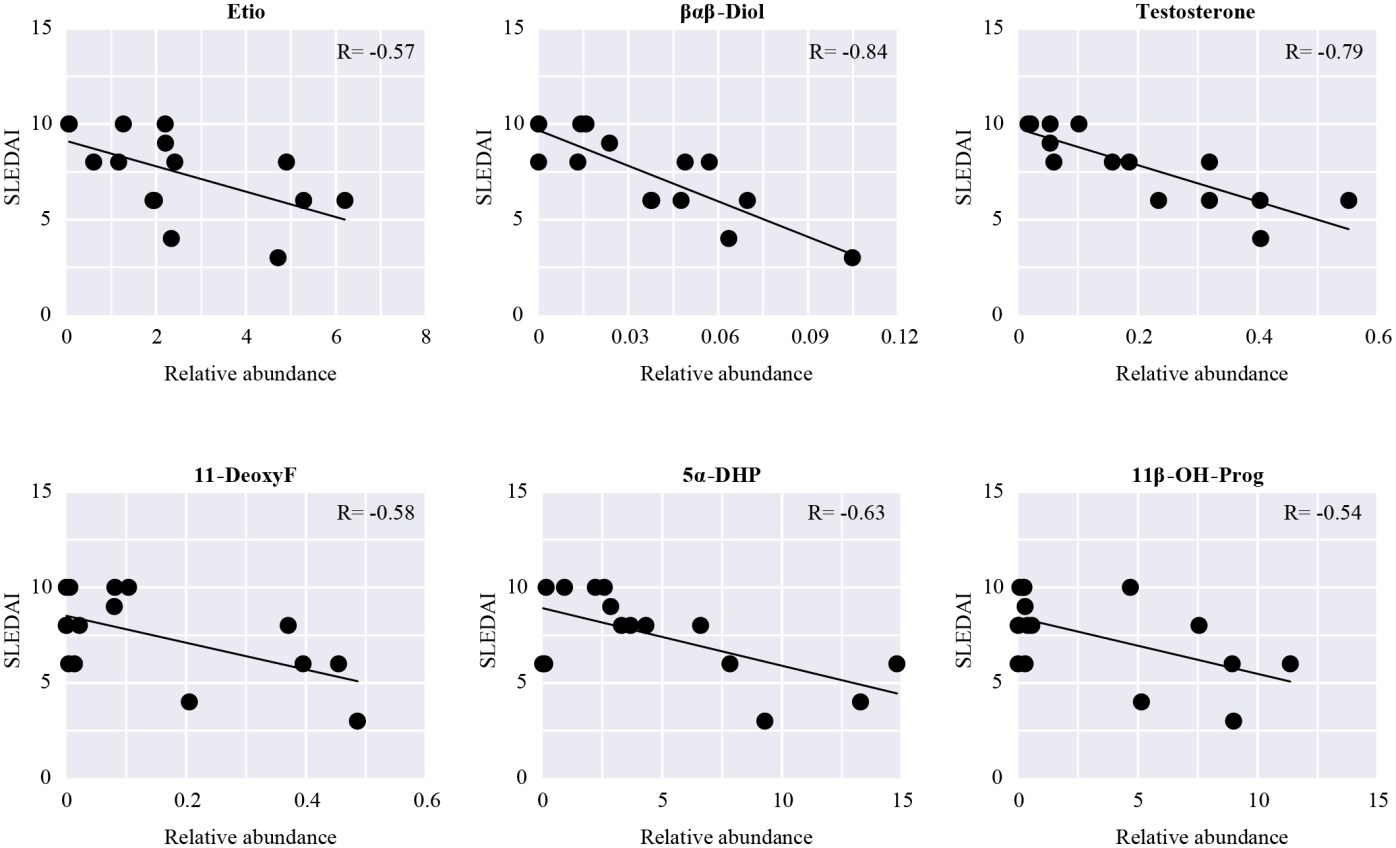
Pearson correlation analysis between SLEDAI and the differential steroids in patients with SLE. Scatter plots show steroids significantly related to SLEDAI (P < 0.05).

SLEDAI is an important indicator to assess the severity and activity of SLE clinically; the higher the score, the greater the activity and the more severe the disease. The relationship between the discriminant steroids and SLEDAI was evaluated using Pearson correlation analysis. Among them, three kinds of androgens (Etio, βαβ-Diol, and testosterone), one kind of corticoid (11-DeoxyF), and two kinds of progestins (5α-DHP and 11β-OH-Prog) were negatively correlated with SLEDAI, indicating that, as the levels of these steroids increase, the SLEDAI tends to decrease (**Figure 6**). The changes in these steroids may reflect aggravation or remission of SLE and may be closely related to the development or pathological process of SLE.

## 4 Discussion

SLE is a prototypical chronic autoimmune disease characterized by massive autoantibody production and systemic inflammatory responses involving multiple organs (22). The interaction of genetic and environmental factors and hormones lead to the imbalance of immune function, which lead to the destruction of target organs by circulating autoantibodies and inflammatory immune cells (23). The prominent gender and age bias in the pathogenesis of SLE suggested that hormones may play a pivotal role in this disease. However, the comprehensive changes of steroid hormones in patients with SLE have not been explored. Herein, a GC/MS-based metabolic profiling analysis was performed to reveal the specific steroid changes related to SLE.

Consistent with previous studies, the total amount of estrogens in patients with SLE had a slight trend of increment in this study. Further species analysis identified the upregulation of three estrogens in SLE: 17-Epi-E3, 17α-E2, and E3. It is generally assumed that estrogen can enhance humoral immune response and accelerate autoimmune disease, such as SLE (24, 25). Clinical studies have reported SLE activity flared up after taking female sex hormones (3). In addition, E2 has been shown to accelerate the immune hyperactivity by enhancing B-cell activity and promoting IL-10 production and to increase the production of immunoglobulin G (IgG) anti-double stranded DNA (dsDNA) antibodies in peripheral blood monocytes of patients with SLE (8). Moreover, the role of estrogen receptor (ER) has been investigated in various models of SLE. Ovariectomized New Zealand Black/White (NZB/W) mice treatment with ERα agonist presented with increased levels of autoantibodies and led to worse mortality than the controls. Inversely, ERα deficiency reduced autoantibodies and glomerulonephritis and improved the survival in spontaneous murine model of lupus (NZB/W F1 mice) (26). Estrogen and ER signaling contribute to the activation of a number of cytokines, contributing to disease pathogenesis and organ pathology in lupus (27). Moreover, estrogen influences T-cell signaling and activation in T cells from patients with SLE. *In vivo* experiments have shown that estrogen downregulated FasL expression in an ER-dependent manner, which is a key molecule in inducing T-cell apoptosis, thereby inhibiting autoreactive T-cell apoptosis, suggesting that estrogen-mediated persistence of autoreactive T cells contributed to autoimmune activity of SLE (28).

Androgens are the precursors of estrogens. This study demonstrated the downregulation of androgens in SLE, not only in the total amount of androgens but also in the levels of nine individual androgens: Etio, βαβ-Diol, testosterone, Epi-An, Epi-DHT, DHEA, 16α-OH-DHEA, A-Diol, and androsterone. Previous studies have also found that women with lupus have lower androgen levels, including testosterone, DHT, DHEA, and DHEA-S (13, 29, 30). Historically, a number of studies have suggested that androgens are protective in SLE. Clinical research studies found that men with hypogonadism are at increased risk of developing SLE (31–33). Whereas in NZB/W mice, the female F1 mice develop severe disease in the first year, but only less than half of male mice developed severely within the same period (5, 34). Mechanisms are complex. Androgens can inhibit B lymphopoiesis and suppress the inflammatory responses of peripheral lymphoid cells through effects on T cells and indirect effects on B cells (35). In addition, androgens can also enhance immune complex clearance (36), a process generally associated with the development of SLE. In addition, low plasma testosterone levels in women may lead to decreased ability of the regulatory T cells to express FoxP3 (37). Treatment of lupus-prone mice with testosterone-like anabolic steroids or DHEA significantly reduced IgG anti-dsDNA antibody and improved survival (38–40). However, effort made on patients with SLE with androgenic compounds got totally inconsistent outcomes (41–45). Hence, much more efforts are still needed.

Then, progestins are also differential hormones in this study. Although the total amount of progestins did not change in lupus, two kinds of progestins (5α-DHP and 11β-OH-Prog) did decease in patients with SLE. Historically, SLE was usually characterized by low progesterone levels, just consistent with this study. In fact, progestins also play roles in the immune system but counteract the pathways affected by estrogen (46). Progesterone can impact CD4^+^T and regulatory T cell differentiation and reduce T-cell proliferation and T-cell–dependent responses and cytokine production. Whereas on B cells, progesterone can reduce antibody production (47). Treatment of female NZB/W mice before onset with continuous progesterone can significantly reduce kidney damage, death, and selective inhibition of pathogenic anti-dsDNA IgG in the kidneys and serum (48). Thus, the immunosuppressive effects of progestins suggested its potential protective role on SLE.

Some strengths and weaknesses in this study should be noted. First of all, we adopted a latest but also most accurate metabolomics approach to detect the steroid hormone species. In addition, this study used urine sample instead of serum sample as a non-invasive measurement improvement. Unavoidably, the small sample size and the study design being a cross-sectional study were all disadvantages. Furthermore, although our study identified steroids that are significantly different in the urine of patients with SLE and control populations, it does not provide an in-depth study of the causal relationship between the steroid metabolism disorders and SLE.

In this study, a GC/MS-based metabolic profiling analysis was performed to reveal the specific changes of steroids in the urine of patients with SLE. Up to 75 kinds of steroids were detected and compared between the controls and patients with SLE. The steroid profile was significantly distinguished in patients with SLE, characterized as the increase of three estrogens and two sterols as well as the decrease of nine androgens, one corticoid, and two progestins. In particular, the changes in androgens were the most significant. In addition to the reported DHEA and testosterone, other androgens such as Etio, androsterone, βαβ-Diol, Epi-An, Epi-DHT, 16α-OH-DHEA, and A-Diol also underwent significant changes. Our study revealed the presence of steroid metabolic disorders in patients with SLE, especially the conversion process of androgens to estrogens. These results may be valuable for further exploration of the pathogenesis of SLE and its potential new treatments.

## Data availability statement

The datasets presented in this study can be found in online repositories. The names of the repository/repositories and accession number(s) can be found below: MTBLS7766 (Metabolights).

## Ethics statement

The studies involving human participants were reviewed and approved by the Second Affiliated Hospital of Zhejiang Chinese Medical University (No. AF-BG-006-1.0). The patients/participants provided their written informed consent to participate in this study.

## Author contributions

JZ designed the project. DW, XZ, and YG performed the experiments. JZ and MY conducted data analysis. LY and DW wrote the manuscript. JZ revised the manuscript. All coauthors have read and agreed to the published version of the manuscript.
