# Supplementary figures and images for: Characteristics of steroid hormones in systemic lupus erythematosus revealed by GC/MS-based metabolic profiling
Source: Front Endocrinol (Lausanne). 2023 Jul 27;14:1164679. doi: 10.3389/fendo.2023.1164679 (PMC10415909; doi:10.3389/fendo.2023.1164679)

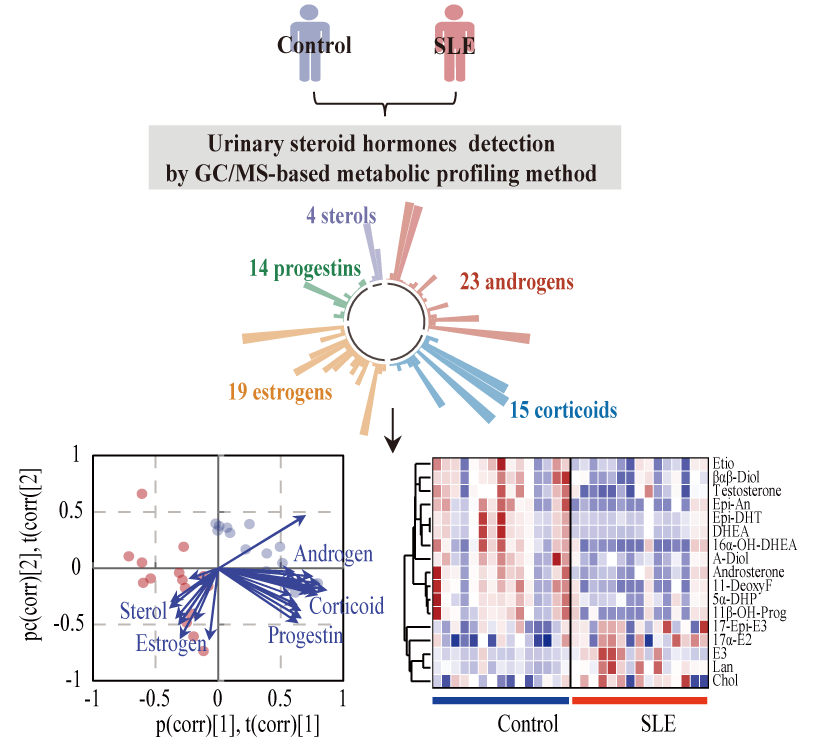

Supplement: Supplementary file 1 [file Image_1.tif]
