# Supplementary material for: Characteristics of steroid hormones in systemic lupus erythematosus revealed by GC/MS-based metabolic profiling
Source: Front Endocrinol (Lausanne). 2023 Jul 27;14:1164679. doi: 10.3389/fendo.2023.1164679 (PMC10415909; doi:10.3389/fendo.2023.1164679)
Supplement: Supplementary file 2 [file Table_1.docx]

Supporting Information (SI) for

**Characteristics of steroid hormones in systemic lupus erythematosus revealed by GC/MS-based metabolic profiling**

**Dehong Wu^1,†^, Lingxia Ye^2,†^, Xiafeng Zhang^3^, Mengdi Yin^3^, Yixuan Guo^3^, Jia Zhou^3, *^**

^1^ Department of Rheumatology, The Second Affiliated Hospital of Zhejiang Chinese Medical University, Hangzhou, Zhejiang, China. ^2^Department of Endocrinology and Metabolism, The Second Affiliated Hospital, Zhejiang University School of Medicine, Hangzhou, Zhejiang, China. ^3^Institute of Basic Research in Clinical Medicine, College of Basic Medical Sciences, Zhejiang Chinese Medical University, Hangzhou, Zhejiang, China.

**^†^**These authors have contributed equally to this work.

**^*^Correspondence:**Jia Zhou
zhoujia@zcmu.edu.cn

This SI contains the following:

Table S1. Clinical characteristics of SLE patients and healthy controls.

**Table S1.** Clinical characteristics of SLE patients and healthy controls

| **Parameter** | **Control (n=15)** | **SLE (n=15)** | **P value** |
| --- | --- | --- | --- |
| Age (years)^a^ | 40.80±2.21 | 41.40±2.63 | 0.86 |
| Female | 15 | 15 | – |
| Disease duration (years) | – | 9.27±1.62 | – |
| Antinuclear antibody positivity | – | 15/15 | – |
| CRP (mg/L) | – | 1.85±0.0.48 | – |
| IgG (g/L) | – | 13.24±0.73 | – |
| Low C3 or C4 | – | 9/15 | – |
| Lupus nephritis | – | 7/15 | – |

^a^Values expressed as the mean ( S. E .); CRP, C-reactive protein; IgG, immunoglobulin; C3, complement C3; C4, complement C4.
