# Supplementary material for: Characteristics of steroid hormones in systemic lupus erythematosus revealed by GC/MS-based metabolic profiling
Source: Front Endocrinol (Lausanne). 2023 Jul 27;14:1164679. doi: 10.3389/fendo.2023.1164679 (PMC10415909; doi:10.3389/fendo.2023.1164679)
Supplement: Supplementary file 3 [file Table_2.docx]

Supporting Information (SI) for

**Characteristics of steroid hormones in systemic lupus erythematosus revealed by GC/MS-based metabolic profiling**

**Dehong Wu^1,†^, Lingxia Ye^2,†^, Xiafeng Zhang^3^, Mengdi Yin^3^, Yixuan Guo^3^, Jia Zhou^3, *^**

^1^ Department of Rheumatology, The Second Affiliated Hospital of Zhejiang Chinese Medical University, Hangzhou, Zhejiang, China. ^2^Department of Endocrinology and Metabolism, The Second Affiliated Hospital, Zhejiang University School of Medicine, Hangzhou, Zhejiang, China. ^3^Institute of Basic Research in Clinical Medicine, College of Basic Medical Sciences, Zhejiang Chinese Medical University, Hangzhou, Zhejiang, China.

**^†^**These authors have contributed equally to this work.

**^*^Correspondence:**Jia Zhou
zhoujia@zcmu.edu.cn

This SI contains the following:

Table S2. Detected steroid hormones by GC-MS.

**Table S2.** Detected steroid hormones by GC-MS

| **Compounds** | **Abbreviation** | **Selected ion** |
| --- | --- | --- |
| **Androgens** |  |  |
| 5β-Androstan-3α ,17α -diol | βαα-diol | 256 |
| 5β-Androstan-3β,17α -diol | ββα-diol | 256 |
| Androsterone | Androsterone | 434 |
| Etiocholanolone | Etio | 434 |
| 5α-Androstan-3α,17β-diol | ααβ-diol | 241 |
| 5β-Androstan-3α,17β-diol | βαβ-diol | 256 |
| 5α-Androstan-3β,17α-diol | αβα-diol | 241 |
| Epidihydrotestosterone | Epi-DHT | 434 |
| 11-Keto-androsterone | 11-Keto-An | 520 |
| 11-Keto-etiocholanolone | 11-Keto-Etio | 520 |
| Dehydroepiandrosterone | DHEA | 432 |
| Epiandrosterone | Epi-An | 419 |
| Androstenediol | A-diol | 434 |
| 5α-Androstanedione | 5α-dione | 432 |
| Epitestosterone | Epi-T | 432 |
| 5α-Androstan-3β,17β-diol | αββ-diol | 241 |
| Dihydrotestosterone | DHT | 434 |
| Androstenedione | A-dione | 430 |
| Testosterone | Testosterone | 432 |
| 11β-Hydroxyandrosterone | 11β-OH-An | 522 |
| 11β- Hydroxyetiocholanolone | 11β-OH-Etio | 522 |
| 16α-Hydroxy-DHEA | 16α-OH-DHEA | 505 |
| Methyltestosterone | Methyl-T | 446 |
| **Corticoids** |  |  |
| Tetrahydrodeoxycortisol | THS | 548 |
| Tetrahydrodeoxycorticosterone | THDOC | 550 |
| Tetrahydrocortisone | THE | 634 |
| Tetrahydrocortisol | THF | 636 |
| Dihydrodeoxycorticosterone | DHDOC | 548 |
| Allotetrahydrocortisol | Allo-THF | 636 |
| 21-Deoxycortisol | 21-deoxyF | 634 |
| 11-Deoxycortisol | 11-deoxyF | 548 |
| 11-Deoxycorticosterone | 11-deoxyB | 516 |
| Cortisone | Cortisone | 615 |
| 11-Dehydrocorticosterone | 11-DehydroB | 617 |
| Allodihydrocorticosterone | Allo-DHB | 636 |
| Allodihydrocortisol | Allo-DHF | 634 |
| Corticosterone | Corticosterone | 634 |
| Cortisol | Cortisol | 632 |
| **Estrogens** |  |  |
| 17α-Estradiol | 17α-E2 | 416 |
| Estrone | E1 | 414 |
| 17β-Estradiol | 17β-E2 | 416 |
| 2-Methoxy-17β-estradiol-3- methylether | 2-Meo-E2-3-methylether | 388 |
| 4-Methoxy-17β-estradiol | 4-Meo-E2 | 446 |
| 2-Methoxyestrone | 2-Meo-E1 | 444 |
| 2-Hydroxy-17β-estradiol-3-Methylether | 2-OH-E2-3-methylether | 446 |
| 2-Methoxy-17β-estradiol | 2-Meo-E2 | 446 |
| 2-Hydroxyestrone | 2-OH-E1 | 502 |
| 2-Hydroxy-17β-estradiol | 2-OH-E2 | 504 |
| 4-Hydroxyestrone | 4-OH-E1 | 502 |
| 4-Hydroxy-17β-estradiol | 4-OH-E2 | 504 |
| 17-Epiestriol | 17-Epi-E3 | 504 |
| Estriol | E3 | 504 |
| 16-Keto-17β-estradiol | 16-Keto-E2 | 487 |
| 16α-Hydroxyestrone | 16-OH-E1 | 487 |
| 16-Epiestriol | 16-Epi-E3 | 504 |
| 2-Hydroxyestriol | 2-OH-E3 | 592 |
| 4-Methoxyestrone | 4-Meo-E1 | 444 |
| **Progestins** |  |  |
| 5β-Dihydroprogesterone | 5β-DHP | 445 |
| Epipregnanolone | Epi-P-one | 447 |
| Pregnanolone | P-one | 447 |
| Allopregnanolone | Allo-P-one | 447 |
| Pregnanediol | P-diol | 269 |
| Pregnanetriol | P-tiol | 435 |
| Pregnenolone | Preg | 445 |
| 5α-Dihydroprogesterone | 5α-DHP | 445 |
| Progesterone | Prog | 458 |
| 20α-Hydroprogesterone | 20α-DHP | 445 |
| 17α-Hydroxypregnenolone | 17α-OH-Preg | 567 |
| 17α-Hydroxyprogesterone | 17α-OH-Prog | 546 |
| 11β-Hydroxyprogesterone | 11β-OH-Prog | 531 |
| 21-Hydroxyprogesterone | 21-OH-Prog | 546 |
| **Sterols** |  |  |
| Cholesterol | Chol | 458 |
| Desmosterol | Des | 441 |
| 24S-Hydroxycholesterol | 24S-OH-Chol | 413 |
| Lanosterol | Lan | 498 |
